# Influence of COVID-19 on dementia caregivers living in community

**DOI:** 10.1093/geroni/igaa057.3477

**Published:** 2020-12-16

**Authors:** Yeji Hwang, Anjali Rajpara, Sonia Talwar, LaShauna Connell, Katie Ramirez, Nancy Hodgson

**Affiliations:** 1 University of Pennsylvania, Philadelphia, Pennsylvania, United States; 2 University of Pennsylvania, Cherry Hill, New Jersey, United States

## Abstract

Community-residing persons living with dementia (PLWD) are a high-risk population for the Coronavirus Disease 2019 (COVID-19) because of advanced age, comorbidities, and impaired cognition. While family caregivers report additional anxiety symptoms by caring for PLWD at home, little is known about their caregiving experience during the current COVID-19 pandemic. The purpose of this study is to examine the influence of the COVID-19 on dementia caregivers. Study participants were recruited from parent study, the Healthy Patterns Clinical Trial (Hodgson; R01NR015226). Data were collected from June 13th to August 28th, 2020 via semi-structured telephone interviews. Thirty-four dementia caregivers participated in this study. Mean caregiver age was 59.2±12.7. Over 70% of the study participant reported worry about spreading COVID-19 to the PLWD and 41.2% reported that they have taken on additional caregiving duties for others in their family or network since COVID-19. In this sample, 61.8% of the dementia caregivers reported one or more anxiety symptoms. Independent t-tests were conducted to compare dementia caregivers who reported anxiety symptoms and those who did not report anxiety symptoms. Anxiety symptoms in dementia caregivers were related to poor physical function of their care recipients (p=0.036). However, anxiety symptoms of dementia caregivers were not related to either cognitive level (p=0.11) or number of neuropsychiatric symptoms of their care recipients (p=0.366). Health care professionals should be alert to the anxiety and concerns expressed by dementia caregivers in order to maintain and improve the well-being of both caregivers and their care recipients during this unprecedented pandemic.

